# Half-dose glucarpidase: an effective rescue for toxic methotrexate plasma concentrations in a patient with bilateral primary vitreoretinal lymphoma

**DOI:** 10.1007/s00277-026-06943-z

**Published:** 2026-04-09

**Authors:** Ángela González-Román, Thais Lizondo, Carla Bastida, Inés Monge-Escartín, Gisela Riu, Esther Carcelero, Eduardo Aguilar, Anna Gaya, Dolors Soy

**Affiliations:** 1 Pharmacy Service, Division of Medicines, Hospital Clínic Barcelona, Barcelona, Spain; 2 Hematology Department, Hospital Clínic Barcelona, Barcelona, Spain; 3 Department of Pharmacology, Toxicology and Therapeutical Chemistry, School of Pharmacy, Universitat de Barcelona, Barcelona, Spain

**Keywords:** Glucarpidase, Methotrexate plasma concentration, Acute kidney injury, Folinic acid

## Abstract

Primary vitreoretinal lymphoma (PVRL) is a rare, aggressive subtype of central nervous system lymphoma that initially presents in the eye. High-dose methotrexate (HD-MTX) is a key component of systemic therapy, but its use can be complicated by nephrotoxicity and delayed drug clearance, requiring urgent management to avoid toxicity. We report the case of a 29-year-old man with bilateral PVRL receiving treatment with the MATRIX regimen. During the fourth cycle, the patient developed acute kidney injury following intravenous HD-MTX (3.5 g/m²), despite prophylactic measures including hydration, urinary alkalinization, and folinic acid rescue. Plasma MTX concentrations remained elevated at 32 µmol/L at 23 hours and 20 µmol/L at 42 hours post-infusion. A reduced glucarpidase dose (25 U/kg), half the standard dose, was administered 50 hours post-infusion, lowering MTX plasma concentrations by 94.6% from the pre-glucarpidase MTX concentration. Intensified folinic acid rescue was maintained. This case demonstrates that MTX clearance may be impaired, highlighting the importance of early therapeutic drug monitoring. Although the standard glucarpidase dose is 50 U/kg, emerging evidence supports the effectiveness of lower doses, which may offer similar clinical benefit while reducing costs. This consideration is particularly relevant in adults, as weight-based dosing substantially increases drug requirements and treatment costs compared to paediatric patients. Low-dose glucarpidase (25 U/kg) proved effective and safe in treating HD-MTX-induced nephrotoxicity in this patient, offering a cost-effective alternative in settings with limited availability of rescue agents, emphasizing the need for timely intervention and multidisciplinary coordination.

## Introduction

Primary vitreoretinal lymphoma (PVRL) is an uncommon and aggressive form of primary central nervous system (CNS) lymphoma that develops within the eye, without initial brain involvement [1, 2]. Although its early symptoms are often mild, PVRL can lead to irreversible vision impairment and has a high risk of spreading to the CNS, which is the main cause of mortality in affected individuals [3, 4].

The management of PVRL includes intravitreal chemotherapy with methotrexate or rituximab as first-line treatment, followed by localized radiotherapy if needed, and systemic chemotherapy regimens with HD-MTX in cases of bilateral involvement or CNS dissemination [5]. In cases of recurrence, intensive chemotherapy followed by autologous stem cell transplantation may be an option [6]. Additionally, newer targeted therapies, such as ibrutinib, temozolomide and lenalidomide are being investigated as potential treatment alternatives [7–9].

## Case presentation

We present a case of a 29-year-old Caucasian man with no relevant medical history, no known allergies or substance abuse. He was diagnosed in March 2024 with bilateral PVRL and was initially treated with the MATRIX regimen, completing three cycles without any incidents. The MATRIX regimen consists of a single dose of rituximab 375 mg/m² and HD-MTX 3500 mg/m² on day 0; cytarabine 2000 mg/m² every 12 h on days 1 and 2; and a single dose of thiotepa 30 mg/m² on day 3 [10].

In August 2024, the patient was electively admitted to the Hematology Department in our hospital to receive the fourth cycle of the MATRIX regimen. On day 0, the patient received intravenous (i.v.) methotrexate (MTX) at a dose of 6570 mg (3.5 g/m²) over a 4-hour infusion. Simultaneously, fluid therapy with 5% Dextrose and 0.9% Sodium Chloride solution (D5NS) was initiated at an infusion rate of 166 mL/h, along with urine alkalinization using an i.v. infusion of 1/6 M (167 mmol/L) sodium bicarbonate commercial solution at 62.5 mL/h. This fluid therapy was maintained at the same infusion rate until discontinuation on day 2. Folinic acid i.v. rescue was started according to hospital protocol, at 75 mg/m² at 12 h post-infusion, followed by 50 mg/m² at 18 and 24 h post-infusion, and then 25 mg/m² every 6 h until plasma MTX concentrations measured at 48 h post-infusion decreased to below 0.1 µM. Monitoring of urinary pH was performed every 6 h to ensure it remained ≥ 7 [10], and corrected with i.v. acetazolamide if required. The pH values that fell below 7 and required rescue therapy are detailed in Fig. 2.

Pre-infusion laboratory tests revealed normal renal and hepatic function with serum creatinine 0.78 mg/dL (reference value (RV): 0.3–1.3 mg/dL), estimated glomerular filtration rate (eGFR), calculated using the CKD-EPI formula [11], higher than 90 mL/min/1.73 m^2^, aspartate aminotransferase (AST) 24 U/L (RV: 5–40 U/L) and alanine aminotransferase (ALT) 57 U/L (RV: 5–40 U/L). However, 24 h after the MTX infusion, the patient developed fever (38.7 °C) along with hypotension (98/48 mmHg), anasarca and facial and periorbital edema (with a 3 kg weight gain). In view of the patient’s clinical deterioration and signs of fluid overload, fluid therapy was discontinued, and empiric antibiotic treatment with i.v. piperacillin/tazobactam and i.v. furosemide was initiated.

**Fig. 1:**
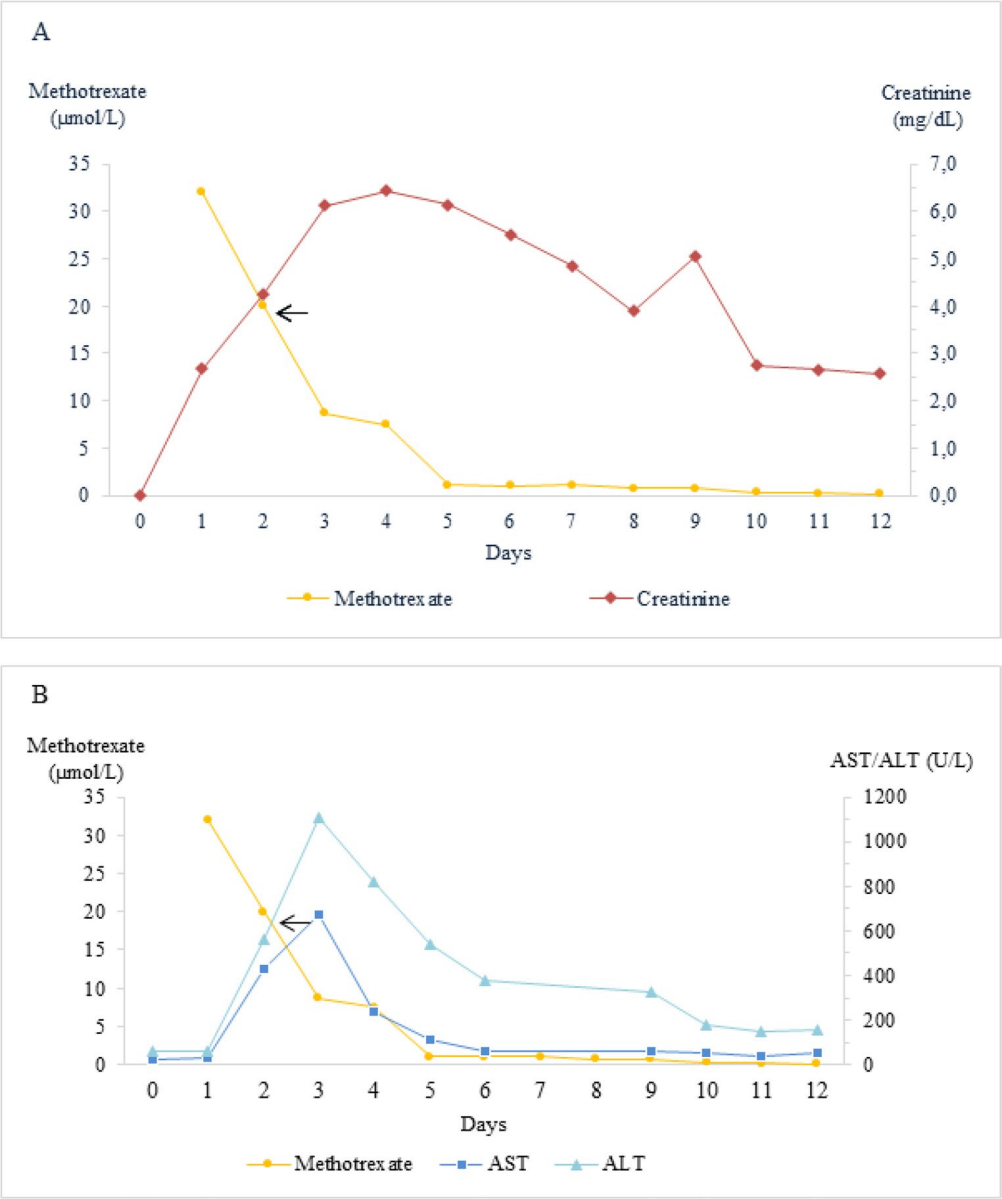
Evolution of the renal and hepatic laboratory values. The horizontal axis indicates the days of follow-up after the start of the infusion and vertical axis indicate the evolution of A) Methotrexate plasma concentration (µmol/L) and serum creatinine (mg/dL); B) Methotrexate plasma concentration (µmol/L), aspartate aminotransferase (AST) and alanine aminotransferase (ALT) values (IU/L). An arrow marks the time point at which glucarpidase was administered

**Fig. 2:**
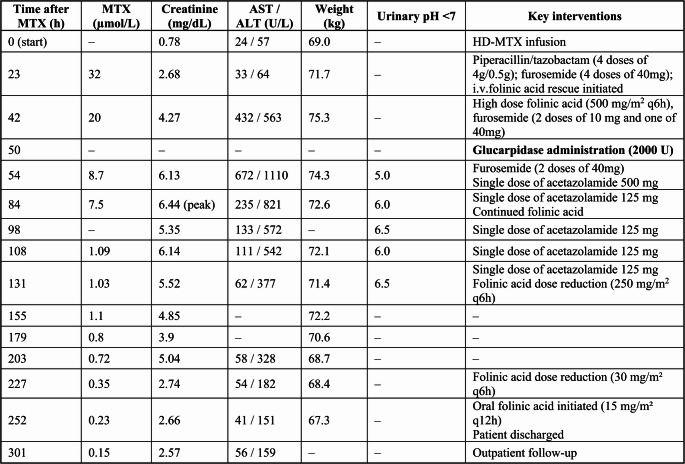
Detailed evolution of plasma methotrexate concentration, renal function, liver enzymes and weight; pH values under 7 and key interventions performed beginning on day 0 of follow-up

Laboratory tests revealed a progressive deterioration of both renal and hepatic parameters. Evolution of laboratory values is depicted in Figs. 1 and 2. Given the suspicion of MTX-induced nephrotoxicity and hepatotoxicity, plasma MTX monitoring was performed, revealing a concentration of 32 µmol/L and 20 µmol/L at 23 and 42 h after start of infusion, respectively. The expected plasma MTX concentrations are < 10 µmol/L at 23 h and < 0.2 µmol/L at 42 h in patients with normal renal clearance [12].

Based on a MTX plasma concentration of 32 µmol/L at 23 h, folinic acid i.v. rescue therapy was intensified to 500 mg/m² every 6 h. As MTX plasma concentration remained at a high concentration at 42 h measurement and renal and hepatic toxicity persisted, glucarpidase administration was indicated [10, 13]. Supportive care continued with i.v. fluids and intensified urinary alkalinization. The patient’s treatment was carefully reviewed for nephrotoxic agents and potential drug interactions, resulting in the discontinuation of piperacillin/tazobactam due to its potential interaction with MTX clearance and acyclovir due to its nephrotoxic risk [14, 15].

Due to the limited in-hospital availability of the drug for urgent use, glucarpidase was administered at a reduced dose of 25 U/kg rather than at the standard 50 U/kg dose as recommended in the national prescribing information [13]. The patient weighed 73 kg, corresponding to a calculated dose of 1825 U. Based on the available presentations, the dose was rounded to 2,000 U, corresponding to two full vials, which was the total administered dose. This dosing strategy was approved by the Hospital’s Special Situations Drug Committee prior to administration. This approach was supported by published case series, almost all conducted in pediatric patients, demonstrating the efficacy of lower-dose glucarpidase in reducing MTX plasma concentrations [10, 16–21]. The enzyme was administered within 50 h of the start of the infusion. The patient received leucovorin at a dose of 500mg/m^2^ five and a half hours before and four hours after glucarpidase administration.

Serial MTX concentration measurements were performed daily after glucarpidase administration, resulting in 8.7 µmol/L at 4 h and 7.5 µmol/L at 36 h after glucarpidase administration, respectively, and bicarbonate solution was subsequently restarted at an infusion rate of 42 mL/h. However, due to the analytical technique used and intracellular MTX release, these values may be falsely elevated during the following 48 h. The first reliable MTX concentration obtained resulted in 1.09 µmol/L 60 h after glucarpidase administration. Evolution of plasma MTX concentrations is shown in Figs. 1 and 2. On day 6 after MTX infusion, four days following glucarpidase administration, plasma MTX concentration decreased to 1.03 µmol/L and folinic acid i.v. rescue was reduced to 250 mg/m^2^ every 6 h. By day 10, plasma MTX had further decreased to 0.35 µmol/L and folinic acid was reduced to 30 mg/m^2^ every 6 h, in accordance with institutional high-dose MTX rescue protocols [22]. The patient was discharged the next day, as soon as the MTX concentrations reached 0.23 µmol/L and treatment with oral leucovorin was continued at a dose of 15 mg/m² every 12 h. Subsequently, MTX concentrations decreased to below 0.2 µmol/L by day 12 and were undetectable (< 0.04 µmol/L) in a control laboratory test performed on day 23, when oral leucovorin was discontinued.

Following glucarpidase administration and continued supportive care, the patient showed progressive clinical and laboratory improvement (Fig. 1) and achieved normalization of renal function parameters within 23 days and liver function parameters within 57 days. The patient was discharged 12 days after chemotherapy administration and was followed up for 9 months with a favourable progression.

The patient was initially scheduled to receive a total of four cycles of the MATRIX regimen. Since the episode of MTX-related toxicity occurred during the fourth and final cycle, no additional high-dose MTX courses were planned or required. Consequently, the toxicity event did not alter the overall treatment plan or result in missed or delayed doses of MTX.

## Discussion

HD-MTX, defined as a single dose ≥ 500 mg/m², is a key component in the treatment of hematologic malignancies and certain solid tumours. Its primary route of elimination is renal, accounting for 70–90% of total clearance [10, 23]. Acute kidney injury (AKI) occurs in up to 12% of patients, impairing methotrexate excretion and increasing the risk of systemic toxicity [24].

Several factors may predispose to MTX-induced nephrotoxicity, including advanced age, baseline renal impairment, and concurrent use of drugs that interfere with MTX clearance (e.g. non-steroidal anti-inflammatory drugs, sulphonamides, penicillins, proton pump inhibitors). Genetic variability in pathways related to MTX transport and metabolism has also been implicated in modifying individual susceptibility to MTX-induced nephrotoxicity. Certain pharmacogenetic polymorphisms may alter drug handling, leading to higher systemic exposure or delayed elimination, thereby increasing the risk of renal adverse effects [24, 25]. Additionally, as a weak acid, MTX precipitates and forms crystals in the renal tubules when the urine is acidic, thereby exacerbating renal injury [26, 27].

At our institution, the concomitant use of penicillins with HD-MTX is not considered a standardized contraindication. Upon admission for HD-MTX, a structured medication reconciliation process is performed, with specific attention to agents more consistently associated with impaired MTX clearance, including proton pump inhibitors, sulphonamides, and nonsteroidal anti-inflammatory drugs. These medications are included in our institutional checklist of “high-alert” agents.

In contrast, piperacillin/tazobactam is not routinely incorporated into this checklist, as its interaction with MTX clearance, although reported in the literature [14], is less consistently characterized compared with the aforementioned drug classes.

In the present case, piperacillin/tazobactam was initiated approximately one hour after HD-MTX administration in response to a febrile episode. The patient received four doses on the same day, after which the antibiotic was discontinued. Currently, our electronic prescribing system generates a specific automated alert to notify clinicians of this drug-drug interactions. However, the medical team evaluated the benefit-risk profile of the administration of this antibiotic and decided to prescribe it.

In addition to concomitant piperacillin/tazobactam exposure, the presence of third spacing may have contributed to delayed MTX clearance in this patient. Within the first 24 h after HD-MTX administration, the patient developed clinically evident edema accompanied by a 3-kg weight gain, suggesting significant fluid redistribution. Third-space fluid accumulation is a well-recognized factor associated with prolonged MTX elimination, as it may serve as a reservoir for delayed drug redistribution back into the intravascular compartment, thereby contributing to sustained plasma concentrations [28].

Supportive care measures, such as folinic acid rescue, hyperhydration (> 3 L/m^2^) and urine alkalinization with acetazolamide in cases of persistent low urinary pH, remain standard. But in cases of delayed MTX clearance with renal dysfunction, glucarpidase might be necessary [10].

Glucarpidase (carboxypeptidase G2) is a recombinant enzyme that rapidly hydrolyzes MTX into inactive metabolites ((2,4-diamino-N10-methylpteroic acid (DAMPA) and glutamate), reducing plasma MTX concentrations by more than 97% within 7–50 min [17]. It is indicated when MTX concentrations exceed 10 µmol/L at 42 h after the start of the MTX infusion, in the presence of renal dysfunction [13]. While the recommended dose is 50 U/kg, various case series in both paediatric and adult populations have shown that lower doses (10–31 U/kg) can achieve comparable efficacy, especially when administered within the optimal time window of 48–60 h post-infusion [10, 16–21]. Furthermore, dose reduction strategies carry significant pharmacoeconomic benefits, addressing a major barrier to widespread glucarpidase use, particularly in adults: its high cost. The approximate cost of a 1,000-U vial of glucarpidase is €19,600; for our 73 kg patient, the standard 50 U/kg dose would require 3,650 U (~ 4 vials, €78,400).

Our case illustrates the effective use of a single half-dose of glucarpidase (25 U/kg) in a patient who developed AKI following HD-MTX therapy. Despite a plasma MTX concentration of 20 µmol/L at 42 h, concentrations decreased following glucarpidase administration.

During the first 48 h after glucarpidase administration, MTX concentrations are reliable only when determined by HPLC, as immunoassays tend to overestimate MTX levels due to cross-reactivity with DAMPA. Given that the half-life of DAMPA is approximately 9–10 h, MTX concentrations measured in our laboratory within this period are expected to be falsely elevated because of assay interference. The enzymatic activity of glucarpidase persists for approximately 18 h, and a rebound in serum MTX concentrations may subsequently occur as the drug redistributes from tissues into the circulation [10, 16–21].

A limitation of our case is the absence of HPLC quantification, which is considered the reference method for accurately measuring MTX concentrations after glucarpidase administration.

Therefore, MTX concentrations obtained by immunoassay during the early post-glucarpidase period should not be used in isolation, given the well-recognized analytical interference caused by DAMPA and other MTX metabolites. As illustrated in our table and figures, the apparently slower decline in MTX concentrations during the first 72 h after glucarpidase administration is likely attributable, at least in part, to immunoassay cross-reactivity rather than true persistence of active drug. In addition, tissue redistribution and/or delayed release from third-space compartments may have contributed to this transient stabilization phase.

In the absence of chromatographic confirmation, early MTX measurements were therefore interpreted with caution and were not used as the sole determinant of therapeutic decision-making. Accordingly, the first reliable MTX measurement was obtained on day 5, more than 48 h after glucarpidase administration. At that time, the concentration was 1.09 µmol/L, representing a 94.6% reduction from the pre-glucarpidase MTX concentration. Although a plateau was observed between 72 and 120 h, consistent with the well-described phenomenon of tissue redistribution, the subsequent decline to 0.8 µmol/L by 144 h supports the effectiveness of the intervention in mitigating life-threatening toxicity and enabling clinical recovery.

This interpretation, based on the overall pharmacokinetic trajectory and the magnitude of reduction rather than on a predefined concentration threshold, provides a more accurate assessment of treatment efficacy. The patient experienced progressive clinical improvement and no adverse effects attributable to glucarpidase.

This favourable outcome underscores the value of early intervention, individualized pharmacological support and multidisciplinary coordination in the management of HD-MTX toxicity. No adverse effects related to glucarpidase were observed in our patient.

In conclusion, a single half-dose of glucarpidase successfully reduced MTX concentrations below the limit of toxicity allowing completion of MTX elimination in 30 days (< 0.04 µmol/L) and recovery from renal and hepatic toxicity. Our findings support the efficacy and safety of low-dose glucarpidase, which also presents a substantial cost-saving strategy, particularly relevant in settings with limited access to high-cost rescue therapies.

## Data Availability

Data sharing is not applicable to this article as no datasets were generated or analyzed during the current study.
